# Identifying the Patterns: Prevalence and Management of Trauma and Surgical Emergencies in a Tertiary Care Hospital in Peshawar

**DOI:** 10.7759/cureus.86462

**Published:** 2025-06-20

**Authors:** Muhammad Raheel, Hameed Ullah, Abdul Haseeb, Muhammad Moeed, Fawad Ali, Uzma Wahid, Sofia Shah, Qaidar Alizai

**Affiliations:** 1 Department of Urology, Institute of Kidney Diseases, Hayatabad Medical Complex, Peshawar, PAK; 2 Department of Surgery, Hayatabad Medical Complex, Peshawar, PAK

**Keywords:** emergency, emergency care, pakistan, peshawar, surgery, trauma

## Abstract

Introduction

Surgical emergencies are considered a significant part of the overall healthcare burden that usually require timely evaluation and urgent intervention to prevent complications and mortalities. The surgical emergency room (SER) is part of the accident and emergency bay that provides care to patients presenting with acute injuries and non-traumatic surgical complaints. The objective of our study was to investigate the patterns of all diseases presenting to the SER and the differences in treatment and outcomes of patients based on trauma vs. non-trauma complaints.

Methods

We performed a prospective descriptive study at the SER from March 1, 2023, to March 15, 2023. Patients of all ages and either gender presenting to the SER with any complaint during the data collection period were included. Patients were stratified into two groups: Group T (trauma) and Group NT (non-trauma). These groups were compared for baseline characteristics and outcomes. Descriptive statistics were used to summarize the findings. Analyses were conducted to compare trauma vs. non-trauma patients in terms of baseline characteristics, management, and outcomes. A p-value less than 0.05 was considered statistically significant.

Results

A total of 550 patients were analyzed, with 97 (17.6%) in Group T and 453 (82.4%) in Group NT. The mean ± SD age was 31.1 ± 17.7 years, with a male predominance (n = 352, 64.0%). Abdominal pain was the most common presentation (n = 172, 31.3%), followed by trauma (n = 97, 17.6%). There was no statistically significant difference in baseline characteristics and comorbid conditions between trauma and non-trauma patients (p > 0.05). However, trauma patients more frequently received intravenous (IV) analgesics (T: 75 [77.3%] vs. NT: 135 [29.8%], p < 0.001) and IV fluids (T: 78 [80.4%] vs. NT: 107 [23.6%], p < 0.001). Non-trauma patients received more IV antibiotics (T: 20 [20.6%] vs. NT: 155 [34.2%], p = 0.012) and oral analgesics (T: 45 [46.4%] vs. NT: 280 [61.8%], p = 0.004). Critical interventions were more frequent in the trauma group, including chest intubation (T: 5 [5.2%] vs. NT: 0, p < 0.001) and endotracheal intubation (T: 7 [7.2%] vs. NT: 2 [0.4%], p < 0.001). There was no significant difference in the rates of major surgeries (p > 0.05). ICU admissions (T: 25 [25.8%] vs. NT: 10 [2.2%], p < 0.001) and mortality (T: 4 [4.1%] vs. NT: 1 [0.2%], p = 0.003) were higher among trauma patients.

Conclusion

This study offers a detailed overview of surgical emergencies in a tertiary care hospital in Pakistan, highlighting patterns that both mirror and differ from international data. Beyond the description of disease presentations, we compare the management strategies and outcomes of patients based on trauma-related versus non-trauma complaints. Our findings may serve as a reference for future research and development of treatment guidelines for surgical emergencies.

## Introduction

Surgical emergencies are considered a significant part of the healthcare burden that requires more timely evaluation and urgent intervention [1]. The surgical emergency room (SER) is part of the accident and emergency bay that provides care to patients presenting with acute injuries and non-traumatic surgical complaints [2]. Treatment and management of patients in the SER directly depend on the presenting complaints and the available resources [3]. Some require quick circulatory resuscitation with intravenous (IV) fluids and blood products or urgent securing of the airway with intubation, while others require urgent pain relief, sepsis control, or major surgical procedures [4]. The proportions of complaints presenting to the SER may differ across different populations, and so do the management modalities used for the care of these patients [4].

The most common complaints reported in these studies are abdominal pain (related to gastrointestinal or renal problems), urogenital complaints (urinary tract infections [UTIs]), skin infections, and musculoskeletal complaints [5]. The most common trauma complaints are related to road traffic accidents, violence (firearm injuries, stab wounds, blunt injuries), and industrial hazards (electric injuries, sharps injuries, etc.) [3,6]. Quality improvement of our care requires thorough and frequent data analysis [2]. Internationally, there have been numerous studies on the topic, giving insights into surgical emergency care as well as comparing the management and outcomes of trauma versus non-trauma patients [7]. In Pakistan, systematic data on the spectrum of surgical emergencies remain limited [8]. The existing literature focuses on isolated disease conditions with minimal emphasis on the broader spectrum of clinical and epidemiological characteristics of patients presenting with surgical emergencies in tertiary care settings [9].

The objective of our study was to investigate the patterns of all diseases presenting to the SER and the difference in treatment and outcomes of patients based on trauma vs. non-trauma complaints. Our study aims to fill existing knowledge gaps and inform clinical decision-making and healthcare planning by characterizing common complaints, diagnoses, and outcomes.

## Materials and methods

Study design, setting, and duration

We performed a prospective descriptive cross-sectional study at the SER of Hayatabad Medical Complex (HMC), a large teaching hospital in Peshawar, Pakistan. The study was carried out over a two-week period from March 1, 2023, to March 15, 2023.

Inclusion and exclusion criteria

We included patients of all ages and either gender presenting to the SER with any complaint at the time of data collection. Patients were excluded if they refused to consent or were dead on arrival.

Sample size and sampling technique

Considering an anticipated prevalence of 61% of visits to the SER, we estimated a minimum required sample size of 366 patients, with a 95% confidence level, 5% margin of error, and a desired power of 80% using the standard proportion formula. The sample size calculation was carried out using the free OpenEpi software [10]. To increase the power of the study, we collected data from 556 patients over the study period.

Employing a non-probability systematic sampling technique, we enrolled every other patient entering the SER and every other trauma patient received in the trauma bay during the data collection period. 

Data collection protocol

After approval from the Institutional Research and Ethical Board (IREB) of HMC and informed consent from the patients or their families, we approached patients for enrollment in the study. A standardized proforma was used to collect data pertaining to biographic details (age, gender), demographic characteristics (residence, education level), and clinical parameters. Particular attention was given to documenting acute presenting complaints, recent hospitalization history, and pre-existing comorbidities including diabetes mellitus, hypertension, chronic kidney disease (CKD), chronic liver disease (CLD), malignancies, ischemic heart disease, and pregnancy status.

We also collected data on treatment delivered, including IV antibiotics, antipyretics, analgesics, IV fluids, central venous pressure (CVP) line placement, airway instrumentation such as intubation, and any procedures conducted in the trauma bay, minor operating theater (OT), or major operating room. Finally, we recorded data on time to treatment initiation and discharge disposition (observation in the ED, discharge without observation, transfer to the major OT, or admission to the floor/ICU).

Stratification

We stratified our patients into two groups: Group T (all patients presenting with both minor and major acute trauma) and Group NT (all other non-trauma patients). These groups were compared for baseline characteristics and outcomes.

Data analyses

Data were analyzed using the Statistical Package for the Social Sciences (SPSS, Version 25; IBM Inc., Armonk, NY). Descriptive statistics were used to summarize our findings, with continuous variables reported as mean ± standard deviation (SD) and categorical variables expressed as frequencies and percentages.

The analyses focused on characterizing the spectrum of diseases, their relative frequencies, and associated demographic factors. Analyses were made to compare trauma vs. non-trauma patients in terms of baseline characteristics, management, and outcomes. The mean ± SD of continuous variables between the two groups were compared using the independent-samples t-test, and the proportions of categorical variables (e.g., gender distribution) were compared using the chi-square test and Fisher's exact test (when any cell value was less than five). For all analyses, a p-value less than 0.05 was considered statistically significant.

## Results

Baseline characteristics and comorbid conditions

In this study, we enrolled 556 patients, out of which six were excluded due to significant missing data. In the 550 patients analyzed, acute trauma cases accounted for 97 (17.6%) while 453 (82.4%) presented with non-trauma complaints. The mean ± SD age was 31.1 ± 17.7 years with male predominance (n = 352, 64.0%). The majority of the patients resided in rural areas (n = 215, 39.1%), and most of the patients had either no education (n = 195, 35.5%) or attended primary school only (n = 175, 31.8%). Chronic conditions were present in a subset of patients, including diabetes mellitus (n = 58, 10.5%), hypertension (n = 52, 9.5%), CKD (n = 18, 3.3%), and CLD (n = 15, 2.7%) (Table 1).

**Table 1:** Baseline Characteristics of All Patients Presenting to the SER SD: standard deviation; SER: surgical emergency room. Continuous variables are reported as mean ± SD unless otherwise specified. Categorical variables are reported as frequency and percentage.

Variable	Subcategory	Frequency (N = 550)	Percentage
Age in years, mean ± SD		31.1 ± 17.7	
Gender	Male	352	64.0
	Female	198	36.0
Urgency status	Not emergency	225	40.9
	Non-general surgical emergency	58	10.5
	General surgical emergency	170	30.9
	Trauma	97	17.6
Residential address	Rural	215	39.1
	Suburban	190	34.5
	Urban	145	26.4
Education level	None	195	35.5
	School only	175	31.8
	Matriculation	80	14.5
	College	50	9.1
	Graduate	45	8.2
	Unknown	5	0.9
Comorbid conditions	Diabetes mellitus	58	10.5
	Hypertension	52	9.5
	Chronic kidney disease	18	3.3
	Chronic liver disease	15	2.7

Distribution by presenting complaints

Overall, the most common presentation was abdominal pain in 172 (31.3%) patients, followed by 97 (17.6%) trauma patients, 68 (12.4%) urogenital complaints, and 63 (11.5%) skin and soft tissue complaints (Table 2). 

**Table 2:** Categorized Presenting Complaints to the SER ENT: ear, nose and throat; LIF: left iliac fossa; LLQ: left lower quadrant; LOC: loss of consciousness; PR: per rectal; RIF: right iliac fossa; RTA: road traffic accident; RUQ: right upper quadrant.

Category	Subcategory/Examples	Frequency (N = 550)	Percentage
Abdominal pain	Generalized, RIF, LIF, hypogastric, epigastric, RUQ, LLQ	172	31.3
Trauma/Injuries	Falls, RTA, sharps, firearm, stab, blunt, burns, dog bite, industrial	97	17.6
Urogenital	Flank pain, urinary symptoms, testicular/scrotal pain/swelling, PR bleed	68	12.4
Skin/Soft tissue	Ulcers, abscess, cellulitis, lacerations, rash, swelling, infections	63	11.5
Musculoskeletal	Back/joint pain, limb pain, shoulder/elbow/knee pain, foot/toe pain	43	7.8
Gastrointestinal	Vomiting, diarrhea, constipation, foreign body ingestion	28	5.1
Postoperative/Care	Stitch removal, wound discharge, dressing changes, postop care	19	3.5
Neurological	Headache, LOC, paraplegia, numbness	14	2.5
Chest/Respiratory	Chest pain, shortness of breath, pneumothorax (implied)	9	1.6
Miscellaneous	Breast complaints, ENT issues, rare trauma (e.g., bomb blast), genital pruritus	37	6.7

Among the 97 trauma patients, 36 (37.1%) were injured due to a fall, and 27 (27.8%) were road traffic accident. Occupational hazards comprised industrial injuries in 8 (8.2%) and sharps injuries in 7 (7.2%) patients, while violence-related trauma was less common, including firearm injuries in 4 (4.1%) and stab wounds in 3 (3.1%) (Table 3). 

**Table 3:** Categorized Trauma Cases Presenting to the SER SER: surgical emergency room.

Category	Frequency (N = 97)	Percentage
Falls and accidents		
History of fall	36	37.1
Road traffic accident	27	27.8
Occupational hazards		
Industrial injury	8	8.2
Sharps injury	7	7.2
Violence/Assault		
Firearm injury	4	4.1
Stab wound	3	3.1
Physical assault	1	1.0
Environmental/Other		
Electrical injury	2	2.1
Explosives injury	1	1.0
Dog bite	2	2.1
Rodent bite	1	1.0
Unclassified		
Unknown	3	3.1
Vegetation	1	1.0

Sub-analyses of trauma vs non-trauma

In our sample, there was no statistically significant difference in the baseline characteristics and comorbid conditions of trauma and non-trauma patients (p > 0.05) (Table 4).

**Table 4:** Comparison of Baseline Characteristics Between Trauma and Non-trauma Patients (N = 550) p-Value <0.05 was considered statistically significant.

Characteristic	Total (N = 550)	Trauma (N = 97)	Non-trauma (N = 453)	p-Value	Test value
Age (years), mean ± SD	31.1 ± 17.7	28.5 ± 16.2	31.8 ± 18.0	0.08	t = 1.75
Gender, n (%)					
Male	352 (64.0%)	67 (69.1%)	285 (62.9%)	0.25	χ² = 1.32
Address, n (%)					
Rural	215 (39.1%)	42 (43.3%)	173 (38.2%)	0.34	χ² = 0.91
Suburban	190 (34.5%)	30 (30.9%)	160 (35.3%)	0.41	χ² = 0.68
Urban	145 (26.4%)	25 (25.8%)	120 (26.5%)	0.89	χ² = 0.02
Education level, n (%)					
None	195 (35.5%)	40 (41.2%)	155 (34.2%)	0.18	χ² = 1.80
School only	175 (31.8%)	25 (25.8%)	150 (33.1%)	0.15	χ² = 2.06
Matriculation	80 (14.5%)	12 (12.4%)	68 (15.0%)	0.49	χ² = 0.48
College	50 (9.1%)	10 (10.3%)	40 (8.8%)	0.63	χ² = 0.23
Graduate	45 (8.2%)	8 (8.2%)	37 (8.2%)	1.00	χ² = 0.00
Unknown	5 (0.9%)	2 (2.1%)	3 (0.7%)	0.20	

However, trauma patients more frequently received IV analgesics (T: 75 [77.3%] vs. NT: 135 [29.8%], p < 0.001) and IV fluids (T: 78 [80.4%] vs. NT: 107 [23.6%], p < 0.001). On the other hand, the non-trauma group had higher rates of IV antibiotics (T: 20 [20.6%] vs. NT: 155 [34.2%], p = 0.012), oral analgesics (T: 45 [46.4%] vs. NT: 280 [61.8%], p = 0.004), and antipyretics (T: 25 [25.8%] vs. NT: 170 [37.5%], p = 0.027) compared to the trauma group.

Critical interventions were also more frequent in the trauma group, including chest intubation (T: 5 [5.2%] vs. NT: 0, p < 0.001), endotracheal intubation (T: 7 [7.2%] vs. NT: 2 [0.4%], p < 0.001), and CVP line placement (T: 5 [5.2%] vs. NT: 1 [0.2%], p < 0.001), with no difference in the rates of major surgeries among the two groups (p > 0.05).

Overall, 310 (56.4%) were discharged home from the SER, 35 (6.4%) required ICU admission, and 5 (0.9%) of the patients died within 24 hours of presentation. Trauma presentations were associated with higher rates of ICU admissions (T: 25 [25.8%] vs. NT: 10 [2.2%], p < 0.001) and higher mortality rates compared to non-trauma presentations (T: 4 [4.1%] vs. NT: 1 [0.2%], p = 0.003) (Table 5). 

**Table 5:** Treatment Received by Trauma and Non-trauma Patients ICU: intensive care unit; IV: intravenous. p-Value <0.05 was considered statistically significant. Significant results (p < 0.05) are highlighted in bold.

Variable	Total, n (%) (N = 550)	Trauma, n (%) (N = 97)	Non-trauma, n (%) (N = 453)	p-Value	Test value
IV opioids	112 (20.4%)	62 (63.9%)	50 (11.0%)	<0.001	OR = 13.8
IV analgesics	210 (38.2%)	75 (77.3%)	135 (29.8%)	<0.001	OR = 8.2
IV antibiotics	175 (31.8%)	20 (20.6%)	155 (34.2%)	0.012	χ² = 6.3
Oral analgesics	325 (59.1%)	45 (46.4%)	280 (61.8%)	0.004	χ² = 8.2
Antipyretics	195 (35.5%)	25 (25.8%)	170 (37.5%)	0.027	χ² = 4.9
IV fluids	185 (33.6%)	78 (80.4%)	107 (23.6%)	<0.001	χ² = 112.5
Blood transfusion	18 (3.3%)	15 (15.5%)	3 (0.7%)	<0.001	OR = 26.7
Critical interventions	45 (8.2%)	30 (30.9%)	15 (3.3%)	<0.001	OR = 13.1
Central venous line	6 (1.1%)	5 (5.2%)	1 (0.2%)	<0.001	OR = 26.0
Endotracheal intubation	9 (1.6%)	7 (7.2%)	2 (0.4%)	<0.001	OR = 18.3
Chest intubation	5 (0.9%)	5 (5.2%)	0 (0%)	<0.001	
Major surgery	50 (9.1%)	10 (10.3%)	40 (8.8%)	0.63	OR = 1.2
Discharge disposition					
Discharged home	310 (56.4%)	12 (12.4%)	298 (65.8%)	<0.001	χ² = 87.3
Admitted to ward	185 (33.6%)	55 (56.7%)	130 (28.7%)	<0.001	χ² = 28.9
ICU admission	35 (6.4%)	25 (25.8%)	10 (2.2%)	<0.001	χ² = 68.4
Other/Transfer	20 (3.6%)	5 (5.2%)	15 (3.3%)	0.31	OR = 1.6
24-hour mortality	5 (0.9%)	4 (4.1%)	1 (0.2%)	0.003	OR = 20.8

## Discussion

This study provides a detailed analysis of the disease patterns among patients presenting to the SER of our tertiary care hospital. Our study sample consisted predominantly of younger male patients belonging to a poor socioeconomic class and mostly residing in rural and suburban areas. Most of these patients presented with non-trauma surgical emergency complaints, while one-fifth of them presented after sharp or blunt trauma. These groups had comparable baseline disease and patient characteristics, but were still significantly different in terms of the treatment received. Our findings highlight significant epidemiological trends, clinical visits, and management of different cases presenting to the SER, with particular emphasis on differences between trauma and non-trauma cases.

Among these 550 patients, 17.6% were trauma and 82.4% presented with non-trauma complaints. The young male predominance of trauma patients in our cohort reflects broader regional patterns, likely influenced by occupational exposure, common road traffic accidents, and sociocultural practices, supporting the results by Ibrahim AO et al., who reported that about 60.9% of trauma-related complaints are most commonly due to road traffic accidents [11]. Common trauma-related injuries included firearm injuries, stab wounds, road traffic accidents, and industrial injuries. In our society and population, all these areas and occupations are highly male-dominated. A study by Murtaza et al. reported that 87.2% of drivers were male and only 12.8% were female [12]. Similarly, Khurshid et al. reported that about 85.3% of fatalities due to road traffic accidents were in male drivers [13]. Interestingly, even the non-trauma presentations were commonly in male patients. This might be attributable to more frequent hospital visits by male patients in our population, secondary to social and cultural norms [8]. This finding may also be directly related to gender-based disparity, where cultural norms limit the tendencies of female patients to seek emergency healthcare in this population, and broader health education may be necessary to overcome this issue. Since our hospital is a large tertiary care public hospital providing care for patients from across the province at minimal cost, most of our patient population is from the lower socioeconomic class from rural and suburban areas. As reported earlier, we saw no difference in the socioeconomic distribution based on the type of presenting complaints. However, whether there are any discrepancies in the care of our patients based on demographic characteristics is yet to be ascertained and remains a topic for further research.

Coming to the disease characteristics, abdominal pain was the most common presenting complaint, consistent with multiple international studies on the topic due to more acute surgical conditions like appendicitis and intestinal obstruction [5]. This was followed by trauma-related injuries, particularly from road traffic accidents and falls, which show similar findings from other middle-income countries [14]. This was reported by the World Health Organization (WHO), which attributes it to substantially limited preventive measures and occupational safety standards [15]. While studies on major emergency surgeries like thoracic surgery show a higher prevalence of sharp injuries (e.g., stab wounds, firearm injuries) [16], our sample consisted of all patients presenting to the trauma bay and thus included a large number of minor blunt injuries. Other common complaints included urogenital issues, skin tissue infections, and musculoskeletal problems. Our study provides an elaborate and up-to-date dataset on the distribution and treatment of these conditions. It may be used as a scaffold for further research and treatment guidelines.

Despite no difference in baseline characteristics, trauma and non-trauma patients received distinct treatment options. Similar to studies reported by Abu-Snieneh et al. and Dilunga et al., IV analgesics, IV fluids, and IV opioids were more frequently administered to our trauma patients [17,18]. The focus of treatment for injured patients is pain relief and securing the ABC (i.e., airway, breathing, and circulation). As expected, endotracheal intubation and blood product transfusion were more common in this group, findings supported by studies from Crewdson et al. (intubation) and Saviano et al. (transfusion) [19,20]. Regarding the management of non-trauma patients, this group was more commonly associated with receiving IV antibiotics, antipyretics, and oral analgesics, as most non-trauma surgical emergencies are related to infections (e.g., appendicitis, urinary tract infections [UTI]). Similarly, Ozen et al. reported that a significant portion of non-trauma patients received IV antibiotics, while Martin reported the same for oral analgesics [21,22]. Our findings support the appropriate treatment measures provided to patients in the SER. Since we did not follow patients beyond the first 24 hours in the hospital, it is imperative to conduct further studies with a longer follow-up to assess outcomes and the appropriateness of our management strategies. As would be expected, chest tube insertion was frequent in trauma patients (to relieve traumatic hemothorax), and other major surgeries were proportionate among the two groups [6]. This negates any injury-related discrepancy in patient care in our settings. Most of our patients were discharged home, as Kazem et al. reported that most surgical emergency patients can be safely managed without the need for hospital admission [23]. Hospital and ICU admissions were more frequent in the trauma group, which was also reported by Ibrahim NA et al. [24]. This correlates with injury severity and equity among our patients. Nonetheless, there was no difference in 24-hour mortality, a finding consistent with the study by Ibrahim NA et al. [24].

This study might have certain important limitations to mention. As a single-center study limited to the SER and trauma bay at a resource-limited setting, our findings may not be generalizable to other settings and patient populations. While we followed our patients for 24 hours in the hospital, a longer follow-up was not included. We also did not analyze in-hospital treatment and outcomes other than mortality and discharge disposition. Finally, broader social and healthcare access factors were not fully explored. Future multicenter, prospective studies are needed to address these gaps, validate our findings, evaluate whether these treatment patterns correlate with injury severity scores, and explore opportunities to standardize trauma management protocols to potentially improve survival rates.

## Conclusions

This study offers a detailed look at surgical emergencies in a tertiary hospital in Pakistan, showing patterns that sometimes mirror and at other times differ from those seen internationally. Other than an elaborate description of disease presentation, we offer to look at the management and outcomes of our patients based on injury-related presenting complaints. Our findings can be used as a reference point for further research on surgical emergencies and treatment guidelines for these patients.
